# A glycoprotein E gene-deleted bovine herpesvirus 1 as a candidate vaccine strain

**DOI:** 10.1590/1414-431X20154243

**Published:** 2015-07-21

**Authors:** M. Weiss, M.C.S. Brum, D. Anziliero, R. Weiblen, E.F. Flores

**Affiliations:** 1 Setor de Virologia, Departamento de Medicina Veterinária Preventiva, Universidade Federal de Santa Maria, Santa Maria, RS, Brasil; 2 Laboratório de Virologia, Curso de Medicina Veterinária, Universidade Federal do Pampa, Uruguaiana, RS, Brasil

**Keywords:** Cattle pathogen, Immunization, Control, Differential vaccine

## Abstract

A bovine herpesvirus 1 (BoHV-1) defective in glycoprotein E (gE) was constructed from a Brazilian genital BoHV-1 isolate, by replacing the full gE coding region with the green fluorescent protein (*GFP*) gene for selection. Upon co-transfection of MDBK cells with genomic viral DNA plus the *GFP*-bearing gE-deletion plasmid, three fluorescent recombinant clones were obtained out of approximately 5000 viral plaques. Deletion of the *gE* gene and the presence of the *GFP* marker in the genome of recombinant viruses were confirmed by PCR. Despite forming smaller plaques, the BoHV-1△gE recombinants replicated in MDBK cells with similar kinetics and to similar titers to that of the parental virus (SV56/90), demonstrating that the gE deletion had no deleterious effects on replication efficacy *in vitro*. Thirteen calves inoculated intramuscularly with BoHV-1△gE developed virus neutralizing antibodies at day 42 post-infection (titers from 2 to 16), demonstrating the ability of the recombinant to replicate and to induce a serological response *in vivo.* Furthermore, the serological response induced by recombinant BoHV-1△gE could be differentiated from that induced by wild-type BoHV-1 by the use of an anti-gE antibody ELISA kit. Taken together, these results indicated the potential application of recombinant BoHV-1 △gE in vaccine formulations to prevent the losses caused by BoHV-1 infections while allowing for differentiation of vaccinated from naturally infected animals.

## Introduction

Bovine herpesvirus 1 (BoHV-1) is an important pathogen of cattle, associated with a variety of clinical manifestations including respiratory disease (infectious bovine rhinotracheitis), genital disorders (infectious pustular vulvovaginitis or infectious pustular balanoposthitis), transient infertility and abortions in cattle (1). BoHV-1 is an enveloped DNA virus belonging to the family *Herpesviridae*, subfamily *Alphaherpesvirinae*, genus *Varicellovirus* (2). BoHV-1 infection is widely distributed around the world, with the exception of a few European countries that have eradicated it. A number of studies have demonstrated the wide distribution of BoHV-1 infection and disease in Brazil (3,4). Like other alphaherpesviruses, BoHV-1 establishes lifelong latent infection in sensory nerve ganglia following acute infection, from which it can be periodically reactivated and transmitted. Thus, latency and reactivation provide adequate means for virus perpetuation in nature (5).

Vaccination has been largely used as one of the strategies to prevent and to reduce the losses associated with BoHV-1 infection (6). Traditional vaccines usually contain attenuated or whole inactivated virus and induce a serological response undistinguishable from that induced by natural infection. The inability to differentiate vaccinated from naturally infected animals impairs control/eradication efforts based on the identification and segregation and/or culling of seropositive animals (7). In this regard, gene-deleted vaccines that allow for serological differentiation - also called differentiating infected from vaccinated animals (DIVA) vaccines - have arisen as alternatives to traditional vaccines (8). Such vaccines have long been used in several European and North American countries (2). In particular, this strategy fits well for herds and/or regions undertaking control/eradication efforts (8). A similar approach was successfully employed to eradicate pseudorabies virus in several countries (9).

The BoHV-1 genome is approximately 138-kb long and encodes around 70 products, of which 10 are envelope glycoproteins. Envelope glycoproteins play important roles in viral biology, pathogenesis and constitute major targets for the host immune system (10). Interestingly, some envelope glycoproteins are non-essential for virus replication in cell culture and *in vivo* and, as such, have been deleted for the production of attenuated and/or antigenically marked vaccine strains (11). The envelope glycoprotein E (gE) has been the target for deletion in the production of antigenically marked vaccines for several herpesviruses such as BoHV-1 (7,12,13) and BoHV-5 (14,15). The choice of gE has relied upon the following reasons: *i*) gE is non-essential for virus replication *in vitro* and *in vivo* and its deletion does not usually significantly reduce the efficiency of virus replication *in vivo* (16); *ii*) gE deletion usually contributes to viral attenuation (11); *iii*) gE deletion does not affect viral immunogenicity (7,11,13), and *iv*) gE is fairly immunogenic, a desirable property for an antigenic marker (12). For these reasons, most BoHV-1 marker vaccines available worldwide contain recombinant gE-negative viral strains (7,17,18).

Efforts to produce commercially viable BoHV-1 marker vaccines have long been reported in Brazil (19,20). A gE-negative BoHV-1 strain was constructed and evaluated in terms of safety, immunogenicity and potential serological differentiation (21,22). More recently, a gE and thymidine kinase double deletion BoHV-5 recombinant strain was constructed and proposed as a candidate vaccine strain (14). Unfortunately, no BoHV marker vaccine is currently available in Brazil. Here, we constructed a gE-deleted strain from a well characterized genital Brazilian BoHV-1 strain (23) as a potential vaccine candidate. This article reports its construction, *in vitro* characterization and preliminary investigations into its immunogenicity and differential serological properties.

## Material and Methods

### Virus strain, cells and plasmid vectors

The Brazilian BoHV-1 strain SV56/90, isolated from preputial swabs and semen of bulls with balanoposthitis (23), was used as the parental virus to construct recombinant viruses. Madin Darby bovine kidney cells (MDBK, ATCC, CCL-22) maintained in Eagle’s Minimum Essential Medium (HiMedia Laboratories, India), supplemented with 10% inactivated and γ-irradiated fetal bovine serum (Nutricell, Brazil), 100 U/mL penicillin and 100 µg/mL streptomycin (Invitrogen, USA) were used in all procedures.

The plasmid vectors used in the construction/recombination procedures included; *i*) a deletion plasmid (pBoHV-1△gE) to introduce the gE deletion in to the BoHV-1 genome and add the green fluorescent protein (*GFP*) marker; *ii*) a plasmid expressing the bovine immediate-early gene ICP0 (bICP0), used as transactivator of the initiation of the transcription of the immediate early genes of the BoHV-1 genome (24), and *iii*) a plasmid expressing the *GFP* gene used for construction of the pBoHV-1▵gE plasmid. The bICP0 plasmid was kindly provided by Dr. Clinton Jones (University of Nebraska at Lincoln, USA).

### Construction of BoHV-1 gE deletion plasmid

**Figure 1:**
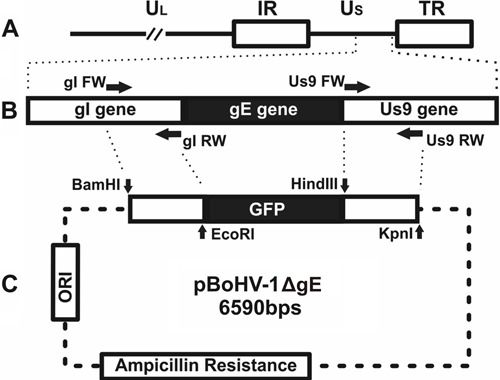
Strategy for the construction of the gE deletion plasmid. *A*, Schematic organization of the BoHV-1 genome and their unique long (U_L_) and short (U_S_) regions and repeats (internal, IR, and terminal, TR). *B*, Amplified view of the organization of the BoHV-1 genome within the U_S_ region corresponding to the *gE* gene. Arrows show the primers used for amplifying the gE flanking regions. *C*, Schematic organization of the deletion plasmid containing the regions for homologous recombination and the restriction endonuclease sites used in the cloning strategy.

The deletion plasmid pBoHV-1▵gE was constructed by replacing the entire gE open reading frame (Figure 1A) with the *GFP* gene as a marker for selection. To construct this plasmid, the upstream and downstream sequences of the *gE* gene (gI and US9, respectively) were amplified by PCR, using Platinum¯ Taq DNA Polymerase High Fidelity (Invitrogen) and cloned into pBlueScriptII KS (+) vector (Stratagene, USA). The gE upstream sequence was PCR amplified using a pair of primers (gI FW: 5′-CACAGGATCCGTTTGTACACAGCTTCGG-3′ and gI RW: 5′-CACAGAATTCTGCCAAATGCCCTTTTCG-3′), resulting in a product of 933 bases pairs (bp) that incorporates *BamH*I/*EcoR*I sites at the 5′ and 3′ ends, respectively. The gE downstream sequence was PCR amplified using a pair of primers (Us9 FW: 5′-CACAAAGCTTCTGTGCCGTCTGACGGAA-3′ and Us9 RW: 5′-CACAGGTACCGCCCGAATCCCCTCCTTC-3′) resulting in a product of 888 bp that incorporates *Hin*dIII/*Kpn*I sites at the 5′ and 3′ ends, respectively (Figure 1B). To introduce the *GFP* gene between the gI and Us9 fragments, a PCR reaction using a pair of primers (*GFP* insertion FW 5′-CACAGAATTCTGACTTGAGCGTCGATTT-3′ and *GFP* insertion RW 5′-CACAAAGCTTCCGATTTCGGCCTATTGG-3′) was performed using the pEGFP-C1 plasmid (Clontech Lab, USA) as template, resulting in a product of 1.856 bp that incorporates *EcoR*I and *Hin*dIII sites at the ends. PCR products were digested with the respective enzymes and cloned between the gI and Us9 fragments. The deletion plasmid (pBoHV-1▵gE) contained the *GFP* gene replacing the *gE* gene (Figure 1C).

### DNA extraction and transfection

Extraction of genomic viral DNA was performed essentially as described (13). Briefly, MDBK cells were inoculated with SV56/90 strain at a multiplicity of infection of 0.1. When the cytopathic effect reached around 90% of the monolayer, the supernatant was collected and clarified by low-speed centrifugation (1,500 *g* for 30 min). The supernatant was then subjected to ultracentrifugation in a 30% sucrose cushion for 2 h at 112,500 *g*. The resulting pellet was resuspended in 1×TE (10 mM Tris-HCl, 5 mM EDTA, pH 8.0) and pre-digested in 1% SDS and RNAse A (Invitrogen) for 30 min at room temperature. The digestion was completed by adding 500 µg/mL of proteinase K (Sigma-Aldrich, USA) and performing a new incubation at 56°C for 30 min. Following digestion, viral DNA was extracted with phenol:chloroform:isoamyl-alcohol (25:24:1), followed by ethanol precipitation according to standard protocols.

DNA of pBoHV-1▵gE and the BoHV-1 bICP0 gene was extracted using the Qiagen Plasmid Midi Kit (Qiagen, USA). Full length viral DNA and plasmids were co-transfected into MDBK cells using Lipofectamine reagent (PolyFect Transfection Reagent, Qiagen) and Opti-Minimum Essential Medium I (Gibco-BRL, USA) as part of the lipofectamine protocol (13,14).

### Generation and selection of recombinant viruses

To generate the BoHV-1 gE deleted virus, the linearized pBoHV-1▵gE plasmid, the full length wild type virus SV56/90 DNA and the bICP0 plasmid were co-transfected into MDBK cells, using Lipofectamine reagent (Invitrogen) as described previously (13). After 48-72 h, cell cultures showing evident cytopathic effects were freeze-thawed, centrifuged at low speed (1,500 *g* for 15 min) and the supernatants were subjected to plaque purification in MDBK monolayers using a low melting agarose overlay. After 72 h, the plates were examined under UV light to search for fluorescent plaques. Fluorescent plaques were picked and amplified in MDBK cells for subsequent characterization.

### PCR confirmation of gE deletion

To confirm deletion of the *gE* gene in the fluorescent viruses recovered from transfected cultures, a PCR reaction using a pair of primers that amplify the deleted region was performed. Total DNA from mock-infected MDBK cells, MDBK cells infected with the parental virus (BoHV-1 SV56/90), or MDBK cells infected with viruses amplified from fluorescent plaques was extracted using proteinase K digestion and phenol/chloroform extraction as described in the section DNA extraction and transfection. The PCR reaction was carried out in a 50-µL volume containing 1 × PCR buffer, 0.2 mM dNTPs, 0.4 µM of each primer (BoHV-1 gE FW: 5′-GCCAGCATCGACTGGTACTT-3′ and BoHV-1 gE RW: 5′-GCACAAAGACGTAAAGCCCG-3′), 1.25 U of Taq DNA polymerase (Invitrogen), 1.5 mM of MgCl_2_, 10% DMSO and 0.1 µg of DNA as template. The PCR conditions consisted of initial denaturation at 95°C for 10 min; followed by 40 cycles of 95°C for 45 s, 57°C for 45 s, 72°C for 1 min and a final extension of 10 min at 72°C. Ten microliters of each reaction were electrophoresed in a 1.5% agarose gel and stained with ethidium bromide. A 325-bp product was expected in DNA samples that contained the *gE* gene. As controls, PCR reactions for the gB coding gene (25) and the *GFP* gene (using the same pair of primers used for the construction of the pBoHV-1▵gE plasmid) were performed.

### Growth properties of recombinants *in vitro*

A virus growth experiment was performed to analyze the kinetics of replication of the BoHV-1▵gE recombinant strain in comparison with the BoHV-1 SV56/90 parental strain. Cultures of MDBK cells were infected with each virus at a multiplicity of infection of 0.1 at 4°C for 1 h. Cultures were then incubated at 37°C with 5% CO_2_, harvested at different intervals and frozen at -80°C. The supernatants were titrated and the titers are reported as TCID_50_/mL (log_10_). To compare plaque size and morphology, MDBK cells were inoculated and adsorbed for 2 h with each virus, overlaid with 1.6% carboxymethylcellulose, incubated for 72 h, fixed with 10% buffered formalin and stained with 0.35% crystal violet.

### Animal inoculation and serological testing

A total of thirteen 2- to 4-month-old male calves, negative for BoHV-1 antibodies, were inoculated with BoHV-1▵gE#3 by the intramuscular route (*im*) at two different doses: 8 animals received a viral dose of 10^7.3^TCID_50_/animal and 5 animals received 10^8.5^TCID_50_/animal. These titers were selected as an average of the titers used by other authors (13,22,26). The number and age of the animals used for each inoculation followed the recommendations set out by the European Pharmacopoeia for tests of BoHV-1 live vaccines. Three calves were inoculated with the parental virus SV56/90 (10^7.3^TCID_50_/animal). The virus neutralization (VN) antibody titers, expressed as the reciprocal of the highest dilution that prevents virus replication, were transformed into geometrical mean titers (GMT-log_2_) (27) for the calculation of the mean antibody titers of each group. After 42 days, sera were tested for virus neutralizing antibodies against BoHV-1 by a VN assay, according to standard protocols (22). To verify seroconversion to gE, serum samples were tested by a commercial anti-gE antibody ELISA test (Bovine Rhinotracheitis Virus gE Antibody Test, IDEXX, The Netherlands). Sera of calves previously inoculated with a gE-positive virus (28) were tested in parallel by a VN assay and an ELISA kit, as additional positive controls in both tests (Table 1).

**Table:**
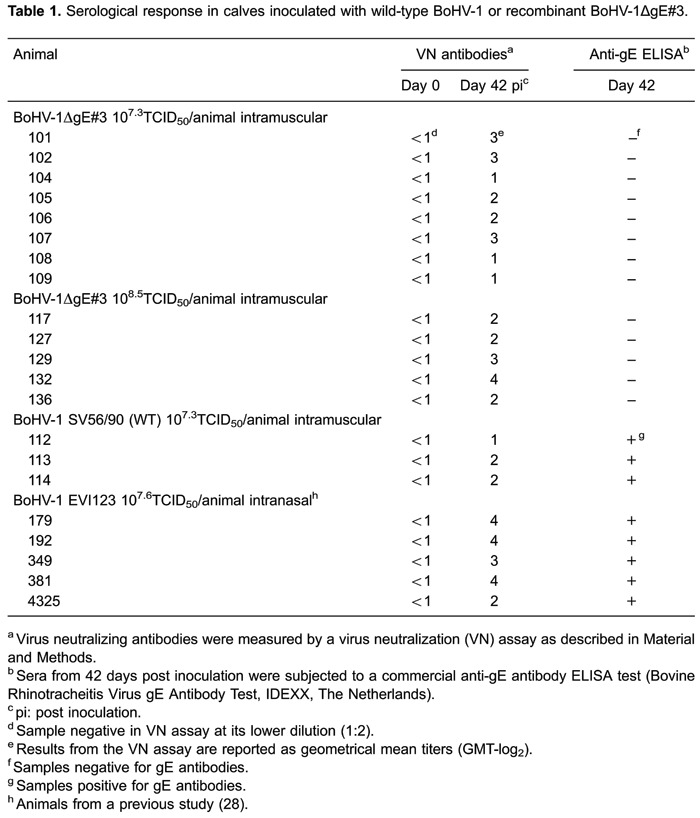

All procedures of animal handling and experimentation were conducted under veterinary supervision and according to the recommendations of the Brazilian Committee of Animal Experimentation (law #6.638 of May 8, 1979). The experiment was approved by an Institutional Animal Ethics Committee, Universidade Federal de Santa Maria (approval #34/2014).

## Results

### Selection of *GFP*-positive, gE-negative BoHV-1 recombinant viruses

**Figure 2:**
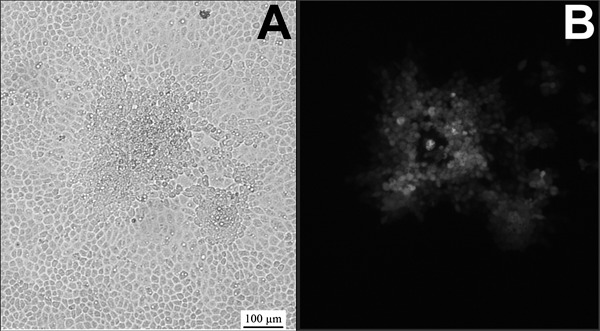
Identification of a recombinant virus expressing the *GFP* selection marker. Plaques formed in MDBK cell monolayers were visualized under light microscopy (*A*) and UV light (*B*).

Recombinant BoHV-1 viruses lacking the *gE* gene were constructed by homologous recombination between genomic DNA of a BoHV-1 strain (SV56/90) and a plasmid containing the gE flanking regions and the *GFP* gene replacing the gE coding region (Figure 1). After two attempts of co-transfection of MDBK cells with parental virus DNA, deletion plasmid and a bICP0 plasmid, and screening of approximately 5000 plaques, three fluorescent plaques were picked and amplified for further characterization. A representative fluorescent plaque is shown in Figure 2. Initially, viral clones derived from the three plaques were amplified and subjected to three rounds of plaque purification.

**Figure 3:**
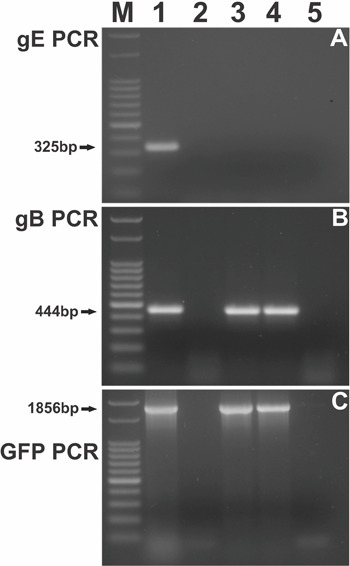
Confirmation of the *gE* gene deletion in the recombinant BoHV-1▵gE by PCR. *A*, PCR of the *gE* gene. *B*, PCR of the *gB* gene. *C*, PCR of the *GFP* marker gene. *Lane M*, molecular weight marker; *lane 1*, parental virus (SV56/90) (*A,B*) or pEGFP-C1 plasmid (*C*); *lane 2*, DNA sample from non-infected MDBK cells - negative DNA control; *lane 3*, BoHV-1▵gE #2; *lane 4*, BoHV-1▵gE #3; *lane 5*, negative control (water).

Then, DNA extracted from MDBK cells infected with each viral clone was subjected to PCR to detect the *GFP* and *gE* genes. One recombinant clone (clone #1) presented double bands in the gE PCR suggesting contamination with a gE-positive virus (data not shown), and was discarded. The remaining clones (#2 and #3) were further characterized. Figure 3 shows that these recombinants were indeed lacking the gE coding region (Figure 3A) and harboring the *GFP* marker gene (Figure 3C). Thus, the recombination strategy was successful and two pure BoHV-1 clones lacking the *gE* gene were obtained. These viral clones were further amplified for characterization and designated BoHV-1▵gE#2 and BoHV-1▵gE#3.

### *In vitro* characterization of BoHV-1▵gE recombinants

**Figure 4:**
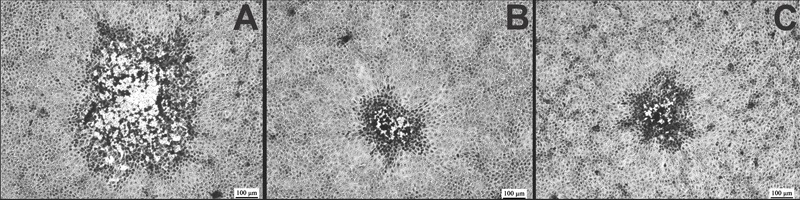
Plaque morphology of the parental virus and the recombinant BoHV-1▵gE viruses. Plaque assays were performed in MDBK cell monolayers overlaid with 1.6% carboxymethylcellulose and stained with crystal violet at 72 h. *A*, Parental virus (SV56/90 strain); *B*, BoHV-1▵gE#2; *C*, BoHV-1▵gE#3.

**Figure 5:**
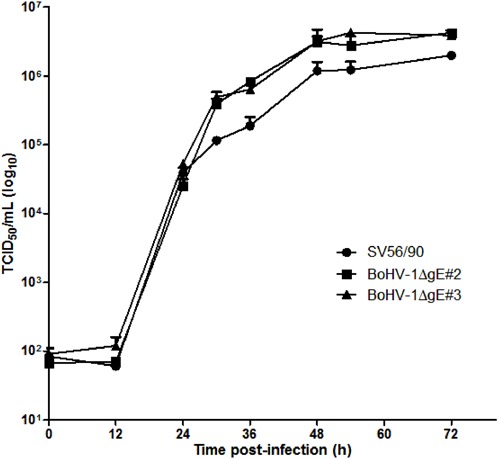
Virus growth curve. MDBK cells were inoculated with the parental virus (BoHV-1 SV56/90) or the recombinant viruses (BoHV-1▵gE clones #2 and #3). At different times post-infection, cells were freeze-thawed and the viral progeny were quantified. The virus titers are reported as log_10_ TCID_50_/mL. Data are reported as means±SE of three different titrations for each time point.

The *in vitro* properties of the recombinants BoHV-1▵gE#2 and #3 were investigated and compared with the parental virus. The plaque size and morphology of the recombinants and parental viruses were monitored in MDBK cell monolayers. In general, the plaques produced by BoHV-1▵gE#2 and #3 were smaller than those produced by the parental virus (Figure 4). The virus growth curve of the recombinants and parental viruses were assayed in MDBK cells and the results are shown in Figure 5. The results demonstrated that both BoHV-1▵gE clones replicated with similar kinetics and to similar - even slightly higher - titers compared with the parental virus. Taken together, these results indicated that gE deletion had no major deleterious effects on the ability of the recombinant viruses to replicate efficiently in cell culture. This is a highly desirable property for a virus intended to be used as a vaccine strain.

### Behavior of the BoHV-1▵gE recombinant *in vivo*: immunogenicity and serological differentiation in calves

As the BoHV-1▵gE recombinant viruses are intended for vaccine use, we next investigated their ability to replicate and to induce an immune response in calves. For this, two groups of calves (eight and five animals each) were inoculated *im* with the BoHV-1▵gE #3 virus in two doses (10^7.3^TCID_50_ and 10^8.5^TCID_50_ per animal) and tested for VN antibodies 42 days after inoculation. All calves inoculated with the BoHV-1▵gE #3 seroconverted, developing VN titers from 2 to 16 at 42 days post inoculation. Similar titers were observed in three calves inoculated with the parental virus (Table 1). These data indicated that the recombinant BoHV-1▵gE#3 is able to replicate efficiently in calves following *im* administration and induce a VN response in levels comparable with that induced by the parental virus.

We next investigated the ability of the recombinant BoHV-1▵gE#3 to induce a serological response that could be differentiated from the immune response induced by a gE positive virus. For comparison, we used serum samples collected from calves inoculated with a wild-type BoHV-1 (28). Serum samples collected at 42 days post inoculation were tested by an anti-gE ELISA kit. As shown in Table 1, all calves immunized with the recombinant BoHV-1▵gE#3 remained negative in the gE ELISA, contrasting with the animals immunized with a gE-positive virus. These data demonstrate that the serological response induced by the recombinant BoHV-1▵gE can be differentiated from that induced by the wild type virus by an anti-gE ELISA test.

## Discussion

A recombinant BoHV-1 with a *gE* gene deletion (BoHV-1▵gE) was constructed with the primary goal of being used as a vaccine strain. Following a long-term trend observed in many European and North American countries, Brazil has also embarked on the development of antigenically marked BoHV-1 strains for vaccine use (19–21). A gE negative BoHV-1 strain constructed from a Brazilian isolate has proven to be safe, immunogenic and allow for serological differentiation (19,21,22). More recently, a double deletion (thymidine kinase/gE) BoHV-5 recombinant was constructed and evaluated positively as a candidate vaccine strain (14,15,28–30). Although BoHV-1 and BoHV-5 are antigenically similar, and vaccines based on either virus are expected to confer cross-protection (28), no vaccine containing gene-deleted BoHV-1 or BoHV-5 is yet available in Brazil. Thus, to address this, we constructed a gE-deleted recombinant BoHV-1 strain. We chose a genital Brazilian BoHV-1 strain (SV56/90) as the parental virus for the following reasons: 1) SV56/90 is a well characterized BoHV-1 strain; 2) it replicates to high titers *in vitro*, a desirable property for a vaccine strain; 3) it is highly immunogenic in cattle; 4) genital and respiratory BoHV-1 are antigenically similar (sometimes undistinguishable) and are highly cross-reactive serologically. Additionally, strain SV56/90 has been extensively characterized at biological, antigenic and molecular levels (31–33).

The strategy of complete *gE* gene deletion has also been used to construct recombinant BoHV-1ΔgE virus by other authors (17,20). Other authors chose to perform a partial deletion of the *gE* gene, keeping the portion next to the *Us9* gene, but no significant differences were observed compared with full deletion (13,26). Using both of these strategies, the possibility of serological differentiation by an ELISA test was maintained.

The homologous DNA recombination that results in the generation of recombinant genomes is a rare event and, as a consequence, the selection of recombinant viruses resulting from this event can be laborious work. The incorporation of the *GFP* gene into the BoHV-1▵gE genome was an easy means to identify and recover gE-deleted recombinant viruses after transfection and also helped to monitor virus purity after plaque purification (13). The bICP0-expressing plasmid was pivotal for the success of the recombination protocol, since bICP0 is an essential transactivator of BoHV-1 immediate early genes (34).

*In vitro* characterization of the two BoHV-1▵gE clones showed that their ability to replicate in cell culture was not adversely affected by *gE* deletion, as they replicated to titers comparable with those of the parental virus (Figure 1). Indeed, previous studies have shown that BoHV-1▵gE recombinants are able to replicate *in vitro* to similar titers as the parental virus (13,14). The ability to replicate to high titers in cell culture is an obvious advantage of virus strains intended to be used for vaccine production. However, the recombinants produced smaller plaques than the parental virus, a property already observed in gE-defective BoHV-1 (7,13) and BoHV-5 viruses (14). This phenotype is probably associated with the fact that gE - complexed with gI - is involved in cell-to-cell spread *in vitro* (35,36). The choice of gE as a target for deletion was also based upon the role of this glycoprotein in anterograde transport of the virus from nerve ganglia to the nose after reactivation of latency (37). Thus, gE-deleted viruses are not transported efficiently back to the nose and, consequently, they are not re-excreted and transmitted upon reactivation (38).

To determine whether the BoHV-1▵gE virus strain would retain its replication ability and immunogenicity *in vivo*, groups of calves were inoculated *im* with the virus and the serological response was measured at 42 days post-inoculation. The serological response of the animal inoculated with the BoHV-1▵gE virus was similar in magnitude to that induced in animals inoculated with wild type virus. In general, the antibody titers observed here were similar to those reported in previous studies (13,26) when live gE-deleted BoHV-1 was inoculated by the *im* route, even when younger animals were used. These results showed that the gE-deleted virus retained its immunogenicity and, thus, has the potential to be used as a vaccine strain. Early studies have shown that gE-deleted herpesviruses generate similar or slightly lower serological responses when compared with wild-type viruses (13) or vaccines strains (7,12).

In addition to the immunogenic potential, an important feature of a gene-deleted vaccine is the possibility of differentiation of vaccinated from naturally infected animals (6). In our testing, the animals inoculated with the recombinant BoHV-1▵gE mounted a serological response that, at 42 days post inoculation, could be differentiated from that mounted by animals inoculated with gE-positive viruses. Although based on a small number of animals, these results demonstrated the differential properties of this candidate vaccine strain.

The *in vivo* data presented here are still preliminary and require further experimentation before the recombinant strain is considered adequate for vaccine use. These studies are underway and include: *1*) safety and immunogenicity tests in different animal categories (including young calves and pregnant cows); *2*) immunogenicity tests using inactivated virus, since the licensing of such vaccines is more feasible in Brazil than with live vaccines; *3*) vaccination-challenge experiments to investigate the ability of the recombinant virus to confer protection upon challenge; and *4*) an experiment to investigate whether the recombinant virus is safe for use in pregnant cows.

An antigenically marked BoHV-1 vaccine to be used in Brazilian cattle would be an important contribution in the control of this infection in a number of ways: *1*) control and eradication of BoHV-1 have been achieved in some European countries using a similar strategy; *2*) Brazil and other South American countries have long been planning to use DIVA vaccines against bovine herpesviruses; *3*) a commercial anti-gE ELISA kit for differentiation of vaccinated from naturally infected animals is already available. For these reasons, and considering the properties demonstrated by recombinant BoHV-1▵gE *in vitro* and *in vivo*, we consider that this strain is suitable to be included in either modified-live or inactivated vaccine formulations for the control of BoHV-1 infection in Brazil.
